# Analyses of the Relation between BPPV and Thyroid Diseases: A Nested Case-Control Study

**DOI:** 10.3390/diagnostics11020329

**Published:** 2021-02-17

**Authors:** Hyo Geun Choi, Young Shin Song, Jee Hye Wee, Chanyang Min, Dae Myoung Yoo, So Young Kim

**Affiliations:** 1 Department of Otorhinolaryngology-Head & Neck Surgery, Hallym University College of Medicine, Anyang 14068, Korea; pupen@naver.com (H.G.C.); weejh07@hanmail.net (J.H.W.); 2 Hallym Data Science Laboratory, Hallym University College of Medicine, Anyang 14068, Korea; joicemin@naver.com; 3 Department of Internal Medicine, CHA Bundang Medical Center, CHA University, Seongnam 13496, Korea; yssongmd@gmail.com; 4 Graduate School of Public Health, Seoul National University, Seoul 08826, Korea; ydm1285@naver.com; 5 Department of Otorhinolaryngology-Head & Neck Surgery, CHA Bundang Medical Center, CHA University, Seongnam 13496, Korea

**Keywords:** benign paroxysmal positional vertigo, thyroid, hypothyroidism, hyperthyroidism, thyroiditis, autoimmune

## Abstract

Background: This study investigated relationship between multiple thyroid disorders and benign paroxysmal positional vertigo (BPPV), adjusting for levothyroxine medication. Methods: The Korean National Health Insurance Service-Health Screening Cohort data from 2002 to 2015 were used. A total of 19,071 patients with BPPV were matched with 76,284 participants of a control group in a ratio of 1:4 for age, sex, income, and region of residence. The previous histories of thyroid disorders such as goiter, hypothyroidism, thyroiditis, hyperthyroidism, and autoimmune thyroiditis were investigated in both the BPPV and control groups. The odds ratios (ORs) for BPPV in thyroid diseases were calculated using conditional logistic regression analyses. Results: The histories of goiter (5.5% vs. 4.1%), hypothyroidism (4.7% vs. 3.7%), thyroiditis (2.1% vs. 1.6%), and hyperthyroidism (3.1% vs. 2.5%) were higher in the BPPV group than in the control group (all *p* < 0.001). Goiter, hypothyroidism, thyroiditis, and hyperthyroidism were associated with BPPV (adjusted OR = 1.28 (95% CI = 1.17–1.39) for goiter, 1.23 (95% CI = 1.10–1.37) for hypothyroidism, 1.13 (95% CI = 1.02–1.26) for hyperthyroidism, each *p* < 0.05). Conclusions: BPPV was associated with thyroid disorders such as goiter, hypothyroidism, thyroiditis, and hyperthyroidism.

## 1. Introduction

The thyroid hormone plays pivotal roles in physical development, neural differentiation, and metabolic regulation [1]. Hypothyroidism is defined as a reduced thyroxine production or secretion from the thyroid gland. The prevalence rate of hypothyroidism is approximately 1–3% in Western general populations [2,3]. In addition to iodine deficiency, autoimmune thyroiditis is known to cause primary hypothyroidism [3]. Increase or a decrease of thyroid hormones induced by autoimmune stimulation was suggested to cause autoimmune thyroid diseases [4]. Hyperthyroidism, a condition with abnormally high levels of thyroxine, could also be caused by thyroid autoantibodies [5]. In iodine-sufficient areas, the overall prevalence of overt hyperthyroidism was estimated to be approximately 0.2% to 1.3%, and approximately 70–80% of hyperthyroidism was Graves’ disease [5,6]. Thyroid dysfunction of hypothyroidism and/or hyperthyroidism has been reported to be associated with other metabolic diseases like diabetes and dyslipidemia, cardiovascular disease, and audiovestibular disorders [7,8,9,10]. A few previous studies have reported the association of vestibular disorders of Ménière disease and benign paroxysmal positional vertigo (BPPV) with thyroid disorders [11,12].

BPPV is one of the most prevalent vestibular disorders, which is estimated to be approximately 10% of the lifetime incidence in the general population [13]. BPPV is characterized by positional vertigo, which is presumed to be caused by dislodged otoconial debris (canalolithiasis) or otoconia attached to the cupula (cupulolithiasis) in the semicircular canals [14,15]. Although a few related conditions, including immobility, head trauma, and inner ear disease the pathophysiologic cause of BPPV remains elusive [16,17,18]. A few previous researchers presumed that hypothyroidism or autoimmune thyroiditis could induce BPPV via diffusion and gravitational stimulation of the thyroid autoantibody-mediated immune complex in the vestibular labyrinth or co-existing microangiitis as autoimmune multi-organ diseases [19,20]. On the other hand, a case-control study showed no significant association of thyroid-stimulating hormone (TSH) and thyroid autoantibody levels with BPPV [21]. The small study population and potential confounders of levothyroxine medication and lifestyle factors could influence the controversial results. Moreover, besides the potential effects of autoimmunity, metabolic disorders in patients with thyroid disorders have been proposed to be related to vertigo and vestibular disorders [22,23]. Thus, various thyroid disorders could have an impact on BPPV. Only a few previous studies reported the association of BPPV with hyperthyroidism and Hashimoto’s thyroiditis [24,25].

We hypothesized that thyroid disorders, including autoimmune thyroiditis and hyperthyroidism, could affect the occurrence of BPPV. To test this hypothesis, the previous histories of thyroid disorders, including goiter, hypothyroidism, thyroiditis, hyperthyroidism, and autoimmune thyroiditis, were analyzed in the BPPV patients compared to control participants adjusting for levothyroxine medication history.

## 2. Materials and Methods

### 2.1. Ethical Considerations

This study was approved by the Ethics Committee of Hallym University (2019-10-023, approval date: 5 November 2019). Written informed consent was waived by the Institutional Review Board. All analyses adhered to the guidelines and regulations of the Ethics Committee of Hallym University. A detailed description of the Korean National Health Insurance Service-Health Screening Cohort data has been described elsewhere [26].

### 2.2. Definition of Benign Paroxysmal Vertigo (Dependent Variable)

Benign paroxysmal vertigo (BPPV) was defined as the diagnosis of ICD-10 codes H811 (Benign paroxysmal vertigo) [27]. We only included participants who were treated ≥2 times by a neurologist or otolaryngologist.

### 2.3. Definition of Thyroid Diseases and Levothyroxine Use (Independent Variable)

The histories of thyroid diseases and levothyroxine use were collected as our previous study [28]. In brief, goiter (E04: Other nontoxic goiters), hypothyroidism (E02 (subclinical iodine-deficiency hypothyroidism) and E03 (other hypothyroidism)), thyroiditis (E06: Thyroiditis), hyperthyroidism (E05: hyperthyroidism), and autoimmune thyroiditis (E063: autoimmune thyroiditis) were defined based on ICD-10 codes and treatment histories 2 or more times. The prescription histories of levothyroxine for ≥3 months were included as a levothyroxine user.

### 2.4. Participant Selection

**Figure 1:**
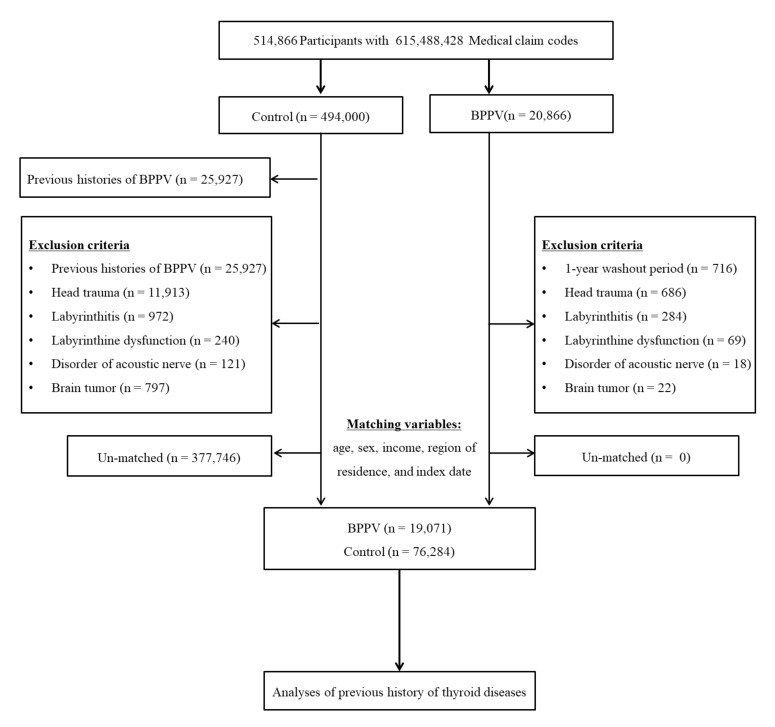
A schematic illustration of the participant selection process that was used in the present study.

BPPV patients were selected from 514,866 participants with 615,488,428 medical claim codes from 2002 through 2015 (*n* = 20,866). The control group was included if participants were not defined as BPPV from 2002 through 2015 (*n* = 494,000). To select the BPPV patient who was diagnosed for the first time, BPPV patients diagnosed in 2002 were excluded (washout periods, *n* = 716). Control participants were excluded if they had previously been diagnosed with BPPV (*n* = 25,927). Participants with a history of treated head trauma (ICD-10 codes: S00 to S09, diagnosed by neurologists, neurosurgeons, or emergency medical doctors) or who underwent head and neck CT evaluations (Claim codes: HA401-HA416, HA441-HA443, HA451-HA453, HA461-HA463, or HA471-HA473) were excluded (*n* = 686 for BPPV, *n* = 11,913 for control). Participants who were treated for labyrinthitis (ICD-10 codes: H811) ≥2 times (*n* = 284 for BPPV, *n* = 972 for control), labyrinthine dysfunction (ICD-10 codes: H832) ≥2 times (*n* = 69 for BPPV, *n* = 240 for control), disorder of the acoustic nerve (ICD-10 codes: H933) ≥2 times (*n* = 18 for BPPV, *n* = 121 for control), and brain tumor (ICD-10 codes: C70 to C72) ≥2 times (*n* = 22 for BPPV, *n* = 797 for control) were excluded. BPPV patients were 1:4 matched with control participants for age, sex, income, and region of residence. To minimize selection bias, the control participants were selected with a random number order. The index date of each BPPV patient was set as the time of BPPV treatment. The index date of the control participants was set as the index date of the matched BPPV patients. Therefore, each BPPV patient matched with control participants had the same index date. During the matching procedure, 377,746 control participants were excluded. Ultimately, 19,071 BPPV patients were 1:4 matched with 76,284 control participants (Figure 1).

### 2.5. Covariates

The age (10 age groups with 5-year intervals), income (5 classes), and region of residence (urban and rural areas) were grouped as our previous study [29]. Tobacco smoking (nonsmoker, past smoker, and current smoker) and alcohol consumption (<1 time a week and ≥1 time a week) status were categorized. Based on the body mass index (BMI, kg/m^2^), underweight (BMI < 18.5), normal (18.5 ≤ BMI < 23), overweight (23 ≤ BMI < 25), obese I (25 ≤ BMI < 30), and obese II (BMI ≥ 30) groups were classified [30]. Missing BMI (47/95,355 (0.049%)) was substituted by the average of the final study groups.

The Charlson Comorbidity Index (CCI) excluding cancer and metastatic cancer was calculated as a continuous variable (0 (no comorbidities) through 29 (multiple comorbidities)) [31,32]. Osteoporosis (M80 to M82) was assigned based on the ICD-10 codes, ≥2 times of treatment histories, and the presence of bone density test using X-ray or CT (Claim code: E7001-E7004, HC341-HC345).

### 2.6. Statistical Analyses

The general characteristics between the BPPV and control groups were compared using the Chi-square test.

To analyze the odds ratios (ORs) with 95% confidence intervals (CIs), a conditional logistic regression model for BPPV in thyroid diseases was calculated. The crude, model 1 (adjusted for obesity, smoking, alcohol consumption, thyroid cancer, osteoporosis, disorders of vestibular function except for BPPV, and CCI scores), and model 2 (additionally adjusted for levothyroxine, goiter, hypothyroidism, thyroiditis, and hyperthyroidism in model 1) were calculated. The analyses were stratified by age, sex, income, and region of residence.

For the subgroup analyses, we divided participants by age and sex (<65 years old and ≥65 years old; men and women) and by income and region of residence (low and high; urban and rural) using crude, model 1, and model 2.

Two-tailed analyses were performed, and significance was defined as *p* values less than 0.05. SAS version 9.4 (SAS Institute Inc., Cary, NC, USA) was used for statistical analyses.

## 3. Results

The rates of thyroid diseases such as thyroid cancer, goiter, hypothyroidism, thyroiditis, and hyperthyroidism were higher in the BPPV group than in the control group, except for autoimmune thyroiditis (Table 1). The rate of levothyroxine medication history was higher in the BPPV group than in the control group. The rate of autoimmune thyroiditis was not different between the BPPV and control groups. Age, sex, income, and region of residence were exactly matched between the BPPV and control groups (*p* = 1.000). The distributions of obesity, smoking status, alcohol consumption, and CCI score were different between the BPPV and control groups (all *p* < 0.001).

The odds for BPPV increased in patients with a history of thyroid diseases, except for autoimmune thyroiditis (Table 2). The OR of BPPV was 1.13 for the history of levothyroxine medication (95% CI = 1.10–1.30). The OR of BPPV was 1.37 for the history of goiter (95% CI = 1.27–1.47). The OR of BPPV was 1.30 for the history of hypothyroidism (95% CI = 1.20–1.40). The OR of BPPV was 1.30 for the history of thyroiditis (95% CI = 1.16–1.46). The OR of BPPV was 1.26 for the history of hyperthyroidism (95% CI = 1.14–1.38). Because thyroid diseases were correlated with each other (Table S1), they were additionally adjusted (model 2). The increased ORs were not changed for the history of goiter, hypothyroidism, thyroiditis, and hyperthyroidism (adjusted OR = 1.28, 95% CI =1.17–1.39 for goiter, adjusted OR = 1.23, 95% CI =1.10–1.37 for hypothyroidism, and adjusted OR = 1.13, 95% CI = 1.02–1.26 for hyperthyroidism). The history of levothyroxine medication was negatively correlated with BPPV after adjusting for other thyroid diseases (adjusted OR = 0.85, 95% CI = 0.75–0.96).

**Figure 2:**
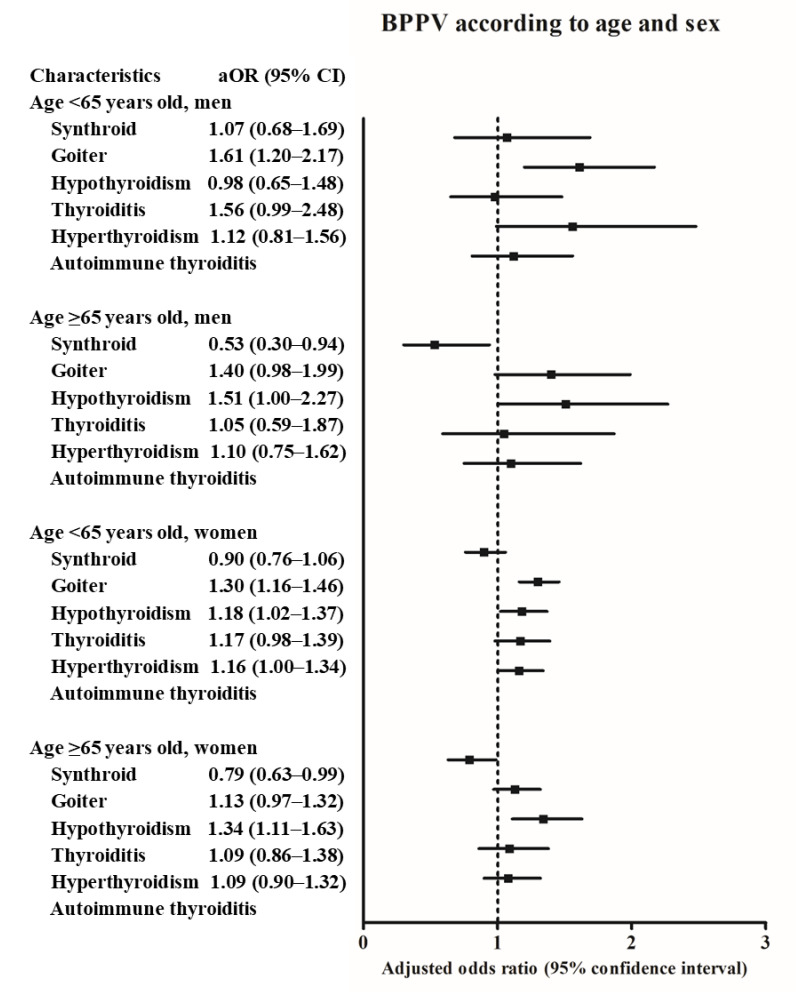
The odds ratios (95% confidence interval) of levothyroxine medication, goiter, hypothyroidism, thyroiditis, hyperthyroidism, and autoimmune thyroiditis for benign paroxysmal positional vertigo according to age and sex.

The associations of thyroid diseases with BPPV differed according to age and sex (Figure 2 and Table S2). The history of goiter was positively associated with BPPV in the <65 years old men subgroup. In the ≥65 years old men subgroup, the history of hypothyroidism was positively related with BPPV. In the <65 years old women subgroup, goiter, hypothyroidism, and hyperthyroidism were positively related with BPPV. In the ≥65 years old women subgroup, the history of hypothyroidism was positively associated with BPPV.

**Figure 3:**
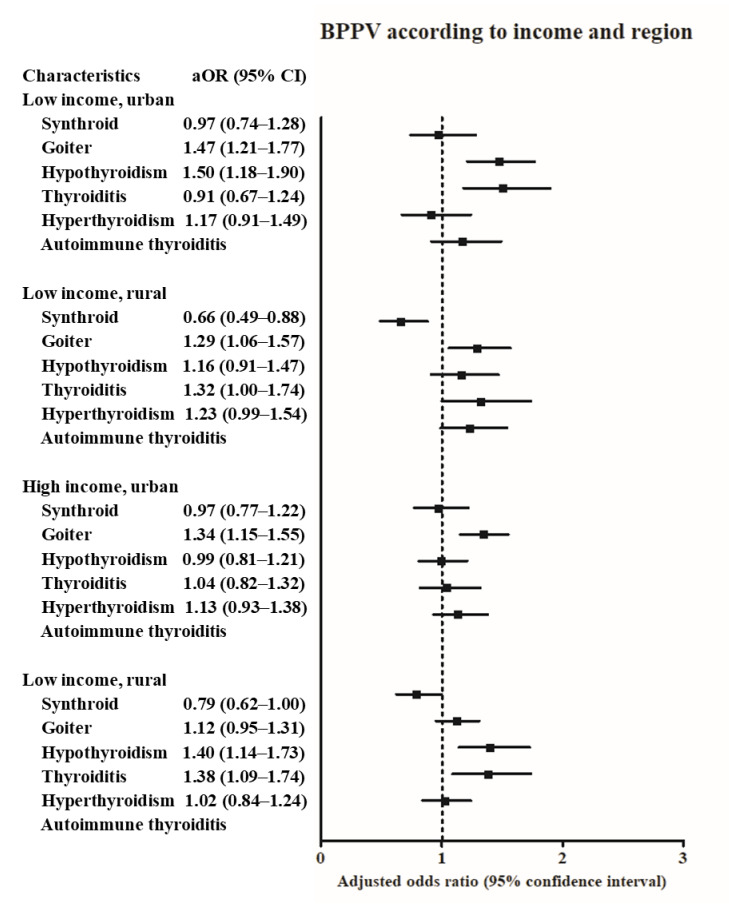
The odds ratios (95% confidence interval) of levothyroxine medication, goiter, hypothyroidism, thyroiditis, hyperthyroidism, and autoimmune thyroiditis for benign paroxysmal positional vertigo according to region of residence.

According to the income and region of residence, the history of goiter was positively related with BPPV in all subgroups except for high income, rural subgroup (Figure 3 and Table S3). The history of hypothyroidism was positively associated with BPPV in the low income, urban subgroup, and high income, rural subgroup. The history of thyroiditis was positively associated with BPPV in the high income, rural subgroup.

## 4. Discussion

Thyroid diseases such as goiter, hypothyroidism, thyroiditis, and hyperthyroidism were related to BPPV in the present study. Both hyper- and hypothyroidism were associated with BPPV. On the other hand, autoimmune thyroiditis did not reveal a statistically significant association with BPPV. According to age and sex, the young women (<65 years old) subgroup showed a higher number of thyroid diseases (goiter, hypothyroidism, and hyperthyroidism) related to BPPV. Although a few prior studies suggested the association of autoimmune thyroiditis with BPPV [20,33] to the best of our knowledge, only a few previous studies have investigated the association of other thyroid diseases with BPPV.

A few previous studies have indicated a relationship between thyroid disease and BPPV [20,33,34]. A case-control study reported a higher rate of hypothyroidism and autoimmune chronic thyroiditis in the BPPV group than in the control group (21% (28/134) vs. 0.02% (2/100) for hypothyroidism and 34% (46/134) vs. 0.02% (2/100) for autoimmune chronic thyroiditis, both *p* < 0.001) [20]. A cross-sectional study demonstrated a higher recurrence rate of BPPV in patients taking thyroid hormone medication (OR = 1.90, *p* = 0.001) [34]. However, in the present study, the history of levothyroxine medication did not show a positive association with BPPV. This could imply that, there is less importance of the status of thyroid function for the risk of BPPV. To support this, a prospective study demonstrated a high rate of BPPV in patients with Hashimoto’s thyroiditis and euthyroid state, compared to healthy control participants (18% (36/ 200) vs. 2% (2/ 200)) [19]. They proposed that thyroid autoantibodies might be crucial for the risk of BPPV [19]. Our previous study demonstrated the association of another cochleovestibular disease, Meniere’s disease, with thyroid diseases of goiter, hypothyroidism, and hyperthyroidism [28]. Thus, the impacts of thyroid diseases on vestibular system could be inferred. Autoimmune dysfunction, dysregulation of endolymphatic flow, and insufficient blood flow of inner ear could mediate the association of thyroid diseases with vestibular dysfunction.

In the present study, autoimmune thyroiditis did not show a significant difference between the BPPV and control groups. The relatively small number of patients with autoimmune thyroiditis could attenuate statistical power. In model 2, autoimmune thyroiditis was not adjusted because it was included in thyroiditis. Although autoimmune thyroiditis was not related to BPPV, thyroiditis was associated with BPPV in this study. Thus, the possible impact of the autoimmune mechanism on BPPV could not be excluded from these present results. Hyperthyroidism and hypothyroidism were positively related with BPPV in this study. Thyroid diseases have common pathophysiology with that of autoimmune response in Graves’ disease and Hashimoto thyroiditis, with genetic or epigenetic susceptibilities [35]. Moreover, levothyroxine medication in patients with hypothyroidism could induce hypothyroidism. For instance, approximately 9.6% of hypothyroidism was attributed to the medication for a previous hyperthyroidism [36]. We analyzed for model 2 to consider the contributions of each thyroid disease on BPPV, in addition to model 1, which analyzed the association of each thyroid disease with BPPV, respectively.

Thyroid autoantibodies could induce autoimmune-complex precipitations in the inner ear, which might change the endolymphatic flow and provoke BPPV. The diffusion of immune-complex of autoimmune thyroiditis patients in the vestibular labyrinth was suggested to increase the risk of BPPV [33]. In addition, the thyroid autoantibody immune complexes in the vestibular system could stimulate the vestibular receptors that evoke vertigo as BPPV [19]. Moreover, autoimmune diseases can manifest in multiple organs of the vestibular labyrinth [33]. Likewise, in the case of thyroid autoantibodies, a case series described the risk of positional vertigo in patients with macroglobulinemia, probably due to the diffusion of macroglobulins in the inner ear [37]. In addition, the association of the autoimmune disease with Vertigo has been suggested [38].

Changes in the endolymphatic ionic composition of the vestibular labyrinth could mediate the BPPV in patients with abnormal thyroid function. It has been suggested that the volume or compositional changes of the endolymph of the vestibular labyrinth could induce BPPV [33,39]. For instance, patients with Pendred syndrome, which is caused by defects in anion exchanger pendrin, often have enlarged vestibular aqueduct (EVA) with recurrent vertigo and hypothyroidism with goiter due to abnormal iodine organification [40,41]. Patients with EVA are known to be prone to BPPV, probably due to the volume or compositional change of the endolymph of the vestibular labyrinth [39,42]. The expression of ion transporters, including sodium iodide symporter and pendrin, could be changed according to thyroid function, which might influence the composition of the endolymph [43,44]. The transcript expression levels of pendrin were reported to be up- and down-regulated in patients with Graves’ disease and Hashimoto thyroiditis, respectively [43]. Therefore, changes in endolymphatic composition could promote the absorption or precipitation of otoconial debris and induce BPPV in patients with abnormal thyroid function.

The impaired microcirculation of blood flow in the inner ear could be linked to the higher rate of BPPV in patients with thyroid disease. It has been reported that the ischemia of the vestibular labyrinth might be one of the predisposing factors for BPPV [45,46]. A case-control study described that the high stimulus rate brainstem auditory evoked potential wave latencies; which is one of the markers for ischemia of the labyrinth, was delayed in BPPV patients compared to those of controls [45]. A prospective study reported that the risk of BPPV was increased in patients with giant cell arteritis compared to controls, probably due to ischemic complications [46]. Moreover, a few clinical studies, including randomized controlled trials [47,48], reported the therapeutic effects of the improvement of blood flow in the inner ear using betahistine in BPPV patients [49]. Several previous studies have suggested an association between abnormal levels of thyroid-related hormones and cardiovascular circulations [50,51]. Some prior studies have proposed increased thyroid-related hormones, including T3 and T4, in conditions of acute systemic ischemia [50]. On the other hand, reduced levels of thyroid-related hormones were also related to cardiovascular compromise [9,51]. Abnormal thyroid function is thought to induce dyslipidemia, endothelial dysfunction, changes in blood pressure, and myocardial dysfunction which elevates the risk of cardiovascular disease and systemic ischemia [9]. Therefore, abnormal thyroid-related hormone levels could reduce blood flow in the inner ear and the risk of BPPV.

This study delineated the association of multiple thyroid disorders, including goiter, hypothyroidism, thyroiditis, hyperthyroidism, and autoimmune thyroiditis, with the incidence of BPPV. Few previous studies have comprehensively investigated the relationship between multiple thyroid diseases and BPPV. Using the large, representative cohort population, the present study demonstrated the positive relationship of each thyroid disease with BPPV. In addition, many comorbidities, lifestyle factors of obesity, smoking, alcohol consumption, and levothyroxine medication were adjusted to attenuate the potential confounding effects. However, this study could not assess the thyroid function and autoimmune antibody status of each participant. Thyroid cancer could not be differentiated according to subtypes. Because microcarcinoma could be diagnosed in Korea by national screening program, the incidence of thyroid cancer may have ethnic differences. However, the proportion of thyroid cancer was small in our cohort, in that the impact of subtypes of thyroid cancer on the association between thyroid diseases and BPPV may not be high. There may have the ethnic difference on the association between thyroid diseases and BPPV, due to the differences on the prevalence of thyroid diseases according to the ethnicity and iodine nutrition [6]. Further, because this study was based on health claims data, patients who did not visit the clinic could be missed. For the diagnosis of BPPV, the types of BPPV could not be differentiated. Future studies are needed to unravel the specific causality between thyroid diseases and the types of BPPV.

## 5. Conclusions

Both hyperthyroidism and hypothyroidism, in addition to autoimmune thyroiditis, were associated with an increased rate of BPPV. The relationship between hypothyroidism and BPPV was consistent according to age, sex, income, or region of residence.

## Figures and Tables

**Table 1:** General Characteristics of Participants.

Characteristics	Total Participants		
	BPPV (*n*, %)	Control (*n*, %)	*p*-Value
Age (years old)			1.000
40–44	250 (1.3)	1000 (1.3)	
45–49	1359 (7.1)	5436 (7.1)	
50–54	2939 (15.4)	11,756 (15.4)	
55–59	3338 (17.5)	13,352 (17.5)	
60–64	3302 (17.3)	13,208 (17.3)	
65–69	3083 (16.2)	12,332 (16.2)	
70–74	2524 (13.2)	10,096 (13.2)	
75–79	1551 (8.1)	6204 (8.1)	
80–84	579 (3.0)	2316 (3.0)	
85+			
Sex			1.000
Male	6878 (36.1)	27,512 (36.1)	
Female	12,193 (63.9)	48,772 (63.9)	
Income			1.000
1 (lowest)	2961 (15.5)	11,844 (15.5)	
2	2295 (12.0)	9180 (12.0)	
3	2875 (15.1)	11,500 (15.1)	
4	4081 (21.4)	16,324 (21.4)	
5 (highest)	6859 (36.0)	27,436 (36.0)	
Region of residence			1.000
Urban	8689 (45.6)	34,756 (45.6)	
Rural	10,382 (54.4)	41,528 (54.4)	
Obesity †			<0.001 *
Underweight	385 (2.0)	1935 (2.5)	
Normal	6392 (33.5)	27,089 (35.5)	
Overweight	5428 (28.5)	20,652 (27.1)	
Obese I	6263 (32.8)	24,098 (31.6)	
Obese II	603 (3.2)	2510 (3.3)	
Smoking status			<0.001 *
Nonsmoker	15,397 (80.7)	60,147 (78.9)	
Past smoker	2003 (10.5)	7047 (9.2)	
Current smoker	1671 (8.8)	9090 (11.9)	
Alcohol consumption			<0.001 *
<1 time a week	14,398 (75.5)	55,586 (72.9)	
≥1 time a week	4673 (24.5)	20,698 (27.1)	
CCI score			
0	12,245 (64.2)	52,632 (69.0)	<0.001 *
1	3376 (17.7)	10,586 (13.9)	
2	1731 (9.1)	6030 (7.9)	
3	804 (4.2)	3040 (4.0)	
≥4	915 (4.8)	3996 (5.2)	
Thyroid cancer	367 (1.9)	298 (1.7)	0.036 *
Osteoporosis	6091 (31.9)	20,328 (26.7)	<0.001 *
Period of taking levothyroxine			<0.001 *
<3month	18,313 (96.0)	73,728 (96.7)	
≥3month	758 (4.0)	2556 (3.4)	
Goiter	1051 (5.5)	3132 (4.1)	<0.001 *
Hypothyroidism	898 (4.7)	2809 (3.7)	<0.001 *
Thyroiditis	400 (2.1)	1241 (1.6)	<0.001 *
Hyperthyroidism	583 (3.1)	1870 (2.5)	<0.001 *
Autoimmune thyroiditis	142 (0.7)	510 (0.7)	0.254

Abbreviations: CCI, Charlson comorbidity index; BPPV, Benign Paroxysmal Positional Vertigo * Chi-square test. Significance at *p* < 0.05. † Obesity (BMI, body mass index, kg/m^2^) was categorized as <18.5 (underweight), ≥18.5 to <23 (normal), ≥23 to <25 (overweight), ≥25 to <30 (obese I), and ≥30 (obese II).

**Table 2:** Crude and adjusted odd ratios (95% confidence interval) for BPPV in levothyroxine, goiter, hypothyroidism, thyroiditis, hyperthyroidism, and autoimmune thyroiditis.

Characteristics	Odd Ratios for BPPV					
	Crude †	*p*-Value	Model 1 †,‡	*p*-Value	Model 2 †,§	*p*-Value
Total participants (*n* = 95,355)						
Levothyroxine	1.20 (1.10–1.30)	<0.001 *	1.13 (1.03–1.24)	0.014 *	0.85 (0.75–0.96)	0.011 *
Goiter	1.37 (1.27–1.47)	<0.001 *	1.31 (1.21–1.42)	<0.001 *	1.28 (1.17–1.39)	<0.001 *
Hypothyroidism	1.30 (1.20–1.40)	<0.001 *	1.24 (1.13–1.35)	<0.001 *	1.23 (1.10–1.37)	<0.001 *
Thyroiditis	1.30 (1.16–1.46)	<0.001 *	1.28 (1.13–1.44)	<0.001 *	1.16 (1.02–1.32)	0.025 *
Hyperthyroidism	1.26 (1.14–1.38)	<0.001 *	1.21 (1.09–1.34)	<0.001 *	1.13 (1.02–1.26)	0.023 *
Autoimmune thyroiditis	1.12 (0.93–1.34)	0.254	1.15 (0.94–1.41)	0.169		

Abbreviations: CCI, Charlson comorbidity index; BPPV, Benign Paroxysmal Positional Vertigo. * Conditional logistic regression model, Significance at *p* < 0.05. † Models stratified by age, sex, income, and region of residence. ‡ Model 1 was adjusted for obesity, smoking, alcohol consumption, thyroid cancer, osteoporosis, disorders of vestibular function, and CCI scores. § Model 2 was adjusted for model 1 plus levothyroxine, goiter, hypothyroidism, thyroiditis, hyperthyroidism.

## Data Availability

Releasing of the data by the researcher is not allowed legally. All of data are available from the database of National health Insurance Sharing Service (NHISS) (https://nhiss.nhis.or.kr/ (accessed on 17 February 2021)). NHISS allows all of this data for the any researcher who promises to follow the research ethics with some cost. If you want to access the data of this article, you could download it from the website after promising to follow the research ethics.

## Supplementary Materials

The following are available online at https://www.mdpi.com/2075-4418/11/2/329/s1, Table S1. Pearson’s chi-square test between each of levothyroxine, goiter, hypothyroidism, thyroiditis, hyperthyroidism, and autoimmune thyroiditis; Table S2. Subgroup analyses of crude and adjusted odd ratios (95% confidence interval) for BPPV in levothyroxine, goiter, hypothyroidism, thyroiditis, hyperthyroidism, and autoimmune thyroiditis according to age and sex; Table S3 Subgroup analyses of crude and adjusted odd ratios (95% confidence interval) for BPPV in levothyroxine, goiter, hypothyroidism, thyroiditis, hyperthyroidism, and autoimmune thyroiditis according to income and region.
